# Sensory neuropathy due to RFC1 in a patient with ALS: more than a coincidence?

**DOI:** 10.1007/s00415-021-10835-9

**Published:** 2021-11-25

**Authors:** Florian Schoeberl, Angela Abicht, Clemens Kuepper, Stefanie Voelk, Stefan Sonnenfeld, Matthias Tonon, Annalisa Schaub, Veronika Scholz, Stephanie Kleinle, Hannes Erdmann, Dieter A. Wolf, Peter Reilich

**Affiliations:** 1 Department of Neurology, Ludwig-Maximilians-University, Marchioninistr. 15, 81377 Munich, Germany; 2 German Center for Vertigo and Balance Disorders, DSGZ, Ludwig-Maximilians-University, Munich, Germany; 3 Department of Neurology, Friedrich-Baur-Institut, Neuromuscular Center, Ludwig-Maximilians-University, Munich, Germany; 4 Medical Genetics Centre, Munich, Germany

Dear Sirs,

In 2019, non-parametric linkage analyses and genome sequencing revealed that biallelic AAGGG expansions in the replication factor C subunit 1 (*RFC1*) gene are a frequent cause of late-onset ataxia [1]. Subsequent studies described the phenotypic spectrum of patients with pathological *RFC1* expansion: they mainly presented in their fifth decade of life with a triad of cerebellar dysfunction (i.e., gait ataxia, dysarthria, ocular motor disorders), sensory neuropathy with concomitant sensory ataxia, and vestibular areflexia bilaterally, denoted by the acronym CANVAS [1]. Quite recently, a multicentre observational study has shown that *RFC1* expansion comprises a multisystemic disease with a chronic dry cough, dysautonomia, and bradykinesia as additional clinical features of variable degree [2].

It is still an outstanding issue, whether biallelic AAGGG expansion in *RFC1* are not associated with an even broader phenotypic spectrum of neurodegenerative diseases.

A 64-year-old male presented with a 1-year history of progressive and painless weakness of both hands. Neurological examination revealed generalized polytopic muscle fasciculations in 4/4 levels, muscle paresis for finger adduction/abduction (r: MRC 4–5, l: MRC 4), finger extension (r: MRC 4–5, l: MRC 4), thumb opposition (r: MRC 4–5, l: MRC 4), wrist extension/flexion (r: MRC 4–5, l: MRC 4) and hip flexion (r/l: MRC 4–5). A split hand sign was conspicuous on both sides. Muscle reflexes were brisk on the left upper limb with decreased ankle jerks bilaterally. There were neither relevant sensory/proprioceptive deficits nor clinical signs of ataxia/vestibulopathy. Clinical suspicion of a degenerative motor neuron disease was confirmed by electromyography, muscle ultrasound and transcranial magnetic stimulation (for details see Table 1). Sural and superficial peroneal nerve potentials and sensory evoked potentials of the tibial nerves (P40) were absent bilaterally.

**Table 1:** An overview of the diagnostic procedures and findings in our patient

Diagnostic test	Result	Interpretation
Muscle ultrasound	Polytopic muscle fasciculations in 4/4 levels	Abnormal
EMG	Acute and chronic denervation in 4/4 levels	Abnormal
Transcranial magnetic stimulation	Delayed central motor latency and reduced amplitude to the left abductor pollicis brevis; normal central motor latencies and amplitudes to the right abductor pollicis brevis and both tibial anterior muscles	Abnormal
Neurofilament light chain serum levels (SIMOA)	82 pg/ml (limit value for ALS: < 45 pg/ml)	Increased
Gold Coast criteria (2020) Awaji-Shima criteria (2008)	Progressive motor impairment, documented by history or repeated clinical assessment, preceded by normal motor function Upper and lower motor neuron dysfunction in at least one body region or lower motor neuron dysfunction in at least two body regions Investigation findings that excluded alternative disease processes Probable ALS: clinical and electrophysiological signs of lower motor neuron degeneration in at least two regions	Fulfilled Fulfilled
Sensory nerve conduction studies	Absent potentials of both sural and superficial peroneus nerves; normal potentials of median and ulnar nerves	Abnormal
Motor nerve conduction studies	Reduced amplitudes of both median and ulnar and left-sided tibial and peroneus nerves; normal potentials of right-sided tibial and peroneus nerves	Abnormal
Sensory evoked potentials	Absent P40 of both tibial nerves; normal N9 and N20 of both median nerves	Abnormal
Caloric irrigation inner ear (warm/cold water °)	Right: warm − 4.8°, Cold 5.7° Left: warm 5.6°, Cold − 7.7° Lying in the range of bilateral presbyvestibulopathy (i.e. 6°–25°)	Abnormal
Video-assisted head impulse-test (median gain at 60 ms)	Right: 0.93 ± 0.11 Left: 0.97 ± 0.06	Unremarkable
MRI-scan brain (3 T)	No pyramidal tract lesion, no brainstem pathology, no cerebellar atrophy, no frontal cortex atrophy	Unremarkable
MRI-scan cervical spine (3 T)	No spinal cord stenosis, no spinal cord lesions, no nerve root compressions	Unremarkable
MRI-scan brachial plexus (3 T)	No lesions, no increased contrast-enhancement, no thickened nerve fascicles	Unremarkable
Additional laboratory testings	Serum glucose, HbA1c-level, liver enzymes, creatinine, vitamin B12 pathway, anti-neuronal antibodies (anti-Hu, -Ri, -Yo, -Ma2, -Tr, Amphiphysin), monoclonal proteins, ganglioside-antibodies (anti-GM1, -GM2, -GD1a, -GD1b, -GQ1b) anti-MAG, antinuclear antibody subtypes (anti-SS-A, -SS-B, -Sm, -RNP, -Scl-70, -PmScl, -Jo1), anti-neutrophilic cytoplasmic antibodies, ganglionic acetylcholine receptor antibodies	Unremarkable
Genetic testings	biallelic AAGGG repeat expansions (~ 400) of the RFC1 locus NGS-based gene panel testing (*ANXA11, CHCHD10, EPHA4, FUS, HNRNPA1, KIF5A, NEK1, OPTN, PFN1, SOD1, TARDBP, TBK1, UBQLN2, UNC13A, VAPB, VCP*) did not reveal variants of unknown significance, pathogenic or likely pathogenic variants (ACMG class 3, 4 or 5) Testing for repeat expansions in *C9orf72* (FTD/ALS), *ATXN1* (SCA1), *ATXN2* (SCA2), *ATXN3* (SCA3), and HTT (Huntington Disease) did not reveal any expansion in the pathological or intermediate range: *C9orf72* (repeat units allele 1/2): 8/8 *ATXN1* (repeat units allele 1/2): 28/29 *ATXN2* (repeat units allele 1/2): 22/22 *ATXN3* (repeat units allele 1/2): 14/30 *HTT* (repeat units allele 1/2): 21/27	Pathological RFC1 repeat expansion ALS gene panel unremarkable Repeat expansions in C9orf72, ATXN1, ATXN2, ATXN3, and HTT unremarkable

Finally, amyotrophic lateral sclerosis (ALS) was diagnosed according to the current diagnostic criteria (see Table 1). Additionally, regarding sensory nerve conduction studies and evoked potentials subclinical sensory neuropathy/neuronopathy was diagnosed.

Acquired conditions for sensory neuropathies/neuronopathies were excluded (see Table 1). The patient did not consent to a recommended additional CSF analysis.

Genetic analysis by CRISPR/Cas9 target enrichment and Oxford Nanopore long-read sequencing [3], revealed biallelic AAGGG repeat expansions (~ 400) of the RFC1 locus. Negative results of all genetic testing are listed in Table 1.

Due to the detected biallelic *RFC1* repeat expansions we post-hoc performed vestibular testing by inner ear calorics and video-assisted head-impulse-test, which revealed isolated bilateral presbyvestibulopathy in the low-frequency range (see Table 1).

This case with a diagnosis of ALS, additional subclinical sensory neuro(no)pathy and bilateral presbyvestibulopathy in the low-frequency range in association with a biallelic *RFC1* expansion raises the following noteworthy future question: is ALS/motor neuron disease within the phenotypic spectrum of biallelic *RFC1* repeat expansions?

To our knowledge, this is the first case of an ALS patient with a concomitant subclinical sensory neuro(no)pathy and bilateral presbyvestibulopathy carrying a biallelic *RFC1* repeat expansion. The number of genes associated with monogenic forms or increased risk of ALS is constantly growing including intermediate expansions of the *SCA 1,2* genes and huntingtin-trinucleotide expansions [4, 5]. Acknowledging previous reports with abnormal findings in sensory nerve conduction studies in up to 20% of patients with ALS [6] and earlier morphological findings in sensory nerve biopsies suggesting loss of sensory root ganglion neurons [7], an involvement of pathological *RFC1* expansions as additional monogenic form or at least genetic risk factor for ALS might be discussed. However, one must admit, that we cannot differentiate an association of pathological *RFC1* expansions with a combined phenotype of ALS and sensory neuro(no)pathy in our patient from a bare coincidence of ALS with a beginning CANVAS phenotype due to *RFC1* pathology. A recent study indeed found *RFC1* expansions exclusively in so far “idiopathic” sensory neuropathies, but not in patients with “idiopathic” sensorimotor neuropathies [8]. Thus a clear link of RFC1 pathology with the motor system is missing so far. Since *RFC1* mediated pathology affects the sensory ganglion cells and not the peripheral sensory nerves, the findings from Currò et al. are plausible [8]. A recent study revealed that pathogenic *SPTLC1* mutations are not only associated with the phenotype of sensory and autonomic neuropathy (i.e. HSAN type 1), but also with juvenile onset ALS [9]. And, for the rare syndrome of “facial onset sensory motor neuronopathy” (FOSMN) typically beginning with sensory symptoms of the trigeminal nerves, underlying TDP-43 pathology in sensory ganglion cells as well as motor neurons was confirmed [10–12], thus classifying FOSMN currently as a rare form of motor neuron disease.

An important limitation of the presented case is, that we cannot assess the influence of rare genetic variants with small effect size or their combinatory effect in terms of polygenic risk modification.

In conclusion, the presented case with a concomitant sensory neuro(no)pathy and proven *RFC1* expansion in addition to ALS should prompt a more systematical search for *RFC1* expansion in larger patient cohorts with ALS and unexplained sensory involvement in order to disentangle a possible role of *RFC1* pathology in ALS.

## References

[1] Cortese A, Tozza S, Yau W et al (2020) Cerebellar ataxia, neuropathy, vestibular areflexia syndrome due to RFC1 repeat expansion. Brain J Neurol [online serial]. https://pubmed.ncbi.nlm.nih.gov/32040566/. Accessed 4 July 2021 10.1093/brain/awz418 PMC7009469 32040566

[2] Traschütz A, Cortese A, Reich S et al (2021) Natural history, phenotypic spectrum, and discriminative features of multisystemic RFC1 disease. Neurology [online serial]. https://pubmed.ncbi.nlm.nih.gov/33495376/. Accessed 4 July 2021 10.1212/WNL.0000000000011528 PMC8055326 33495376

[3] Gilpatrick T, Lee I, Graham JE (2020). Targeted nanopore sequencing with Cas9-guided adaptor ligation. Nat Biotechnol.

[4] Li P, Sun X, Xia G et al (2016) ATXN2-AS, a gene antisense to ATXN2, is associated with spinocerebellar ataxia type 2 and amyotrophic lateral sclerosis. Ann Neurol [online serial]. https://pubmed.ncbi.nlm.nih.gov/27531668/. Accessed 4 July 2021 10.1002/ana.24761 PMC6555153 27531668

[5] Dewan R, Chia R, Ding J et al (2021) Pathogenic huntingtin repeat expansions in patients with frontotemporal dementia and amyotrophic lateral sclerosis. Neuron [online serial]. https://pubmed.ncbi.nlm.nih.gov/33242422/. Accessed 4 July 2021 10.1016/j.neuron.2020.11.005 PMC7864894 33242422

[6] Pugdahl K, Fuglsang-Frederiksen A, de Carvalho M (2007). Generalised sensory system abnormalities in amyotrophic lateral sclerosis: a European multicentre study. J Neurol Neurosurg Psychiatry.

[7] Heads T, Pollock M, Robertson A, Sutherland W, Allpress S (1991) Sensory nerve pathology in amyotrophic lateral sclerosis. Acta Neuropathol (Berl) [online serial]. https://pubmed.ncbi.nlm.nih.gov/1662002/. Accessed 15 July 2021 10.1007/BF00308818 1662002

[8] Currò R, Salvalaggio A, Tozza S et al (2021) RFC1 expansions are a common cause of idiopathic sensory neuropathy. Brain J Neurol [online serial]. 2021. https://pubmed.ncbi.nlm.nih.gov/33969391/. Accessed 26 Sept 2021 10.1093/brain/awab072 PMC8262986 33969391

[9] Jo J, R C, De M et al (2021) Association of variants in the SPTLC1 gene with juvenile amyotrophic lateral sclerosis. JAMA Neurol [online serial]. https://pubmed.ncbi.nlm.nih.gov/34459874/. Accessed 26 Sept 2021 10.1001/jamaneurol.2021.2598 PMC8406220 34459874

[10] Vucic S, Tian D, Chong PST, Cudkowicz ME, Hedley-Whyte ET, Cros D (2006). Facial onset sensory and motor neuronopathy (FOSMN syndrome): a novel syndrome in neurology. Brain.

[11] Vucic S (2014) Facial onset sensory motor neuronopathy (FOSMN) syndrome: an unusual amyotrophic lateral sclerosis phenotype? J Neurol Neurosurg Psychiatry [online serial]. https://pubmed.ncbi.nlm.nih.gov/24643461/. Accessed 26 Sept 2021 10.1136/jnnp-2014-307756 24643461

[12] Rossor A, Jaunmuktane Z, Rossor M, Hoti G, Reilly M (2019) TDP43 pathology in the brain, spinal cord, and dorsal root ganglia of a patient with FOSMN. Neurology [online serial]. https://pubmed.ncbi.nlm.nih.gov/30700593/. Accessed 26 Sept 2021 10.1212/WNL.0000000000007008 PMC6404468 30700593

